# Bridging the Gap: Designing Medical Integration Curricula for Foreign Healthcare Graduates in the Netherlands

**DOI:** 10.5334/pme.1994

**Published:** 2026-03-18

**Authors:** Eva Stortelder, Fokie Huizenga, Iris Homan, Hodayseh Miaei, Richard Horenberg, Annet van Royen-Kerkhof, Harold V. M. van Rijen, Joyce L. Browne

**Affiliations:** 1 Centre of Education and Training, University Medical Centre Utrecht, Utrecht University, Utrecht, The Netherlands; 2 Department of Global Public Health & Bioethics, Julius Centre for Health Sciences and Primary Care, University Medical Centre Utrecht, Utrecht University, The Netherlands; 3 Department of Pediatric Surgery, Queen Fabiola Children’s Hospital, University of Brussels, Belgium; 4 Pediatric Immunology and Rheumatology, Wilhelmina Children’s Hospital, Utrecht, The Netherlands; 5 Graduate School of Life Sciences, Utrecht University, The Netherlands; 6 Utrecht University Centre for Global Challenges (UGlobe), Faculty of Law, Economics and Governance, Utrecht University, The Netherlands

## Abstract

**Background and Rationale for Innovation:**

Each year, numerous refugees with medical training are forced to migrate. Although refugee healthcare professionals are generally highly motivated to continue practicing their professions, they face substantial barriers when integrating into the host country health systems. Given the Dutch healthcare system’s urgent healthcare workforce shortages and lack of diversity, a bridging program to facilitate integration offers mutual benefits. Yet, to date, few integrated curricula for newcoming healthcare professionals exist or have been evaluated.

**Aim and Developmental Approach of Innovation:**

This article presents the development of a bridging module -an educational innovation- designed by an academic hospital to support newly arrived professionals in entering the Dutch healthcare system. The process followed the eight steps of Design-Based Research in Medical Education.

**Outcome:**

Between 2022 and 2024, two pilot refugee observerships and a health systems’ analysis – including a scoping review – led to a six months integration curriculum for newcoming healthcare professionals held in 2024 and 2025. The innovative program received an overwhelming number of applications. Participants reported improved integration and language skills, which supported entry-level employment opportunities. The second edition expanded regionally, with participation of two additional hospitals.

**Conclusion:**

Bridging curricula, coordinated by hospitals, medical faculties and their stakeholders can accelerate the integration of motivated and skilled newly arrived professionals into host country health systems through training and practice. Such initiatives respond to societal needs for a sustainable, adequately staffed, inclusive and diverse healthcare system.

## Background and Rationale for Innovation

By May 2024, 120 million individuals were forcibly displaced worldwide – the highest number on record [1]. While exact figures are not known, this group includes healthcare professionals and medical students, eager to continue their careers and seeking educational or employment opportunities in their new country of residence [2,3]. Despite increasing and multisector labor shortages in Western Europe, a mismatch between qualifications of status holders and the jobs available to them exists. Most secure lower-skilled employment [4], with highly qualified refugees often experiencing a ‘career rewind’ [5]. Different terminology is used to describe people that flee or migrate to another country. Table 1 explains terminology in this paper.

**Table 1:** Explanation of used terminology.


TERMINOLOGY USED	DEFINITIONS

***Foreign healthcare graduates***	Healthcare professionals trained abroad. Term used by the organization ‘CIBG’ of the Dutch ministry of health, involved in healthcare provider registration in the Netherlands*

***Refugees***	Persons unable to return to their country of origin, because of persecution to their race, religion, nationality or political opinion, falling under the refugee convention of the United Nations [1], which includes the core principle of non-refoulement

***Asylum seekers***	Refugees applying for protection in a specific country

***Status holders***	Asylum seekers who have been officially recognized with a residence permit of 5 years

***Labor migrants***	Persons that voluntarily choose to come to a certain country i.e. to work, for education, or marriage

***Newcomers***	Persons that have recently arrived in a country as migrants or asylum seekers. **For this innovation the term Newcomers is referring to status holders only**


*CIBG *Centraal Informatiepunt Beroepen Gezondheidszorg*, Central Information point Healthcare Professions, responsible for BIG-registration. BIG: *Beroepen Individuele Gezondheidszorg*, Professions in individual healthcare registration.

In the Netherlands, approximately 30.000 asylum seekers are granted a residence permit annually [6,7,8]. A substantial proportion of these status holders is eligible for labor market participation, including healthcare professionals. National organizations engaged in refugees’ labor education and labor market integration, such as the University Asylum Fund, report refugee healthcare workers are highly motivated to continue working in their professions. However, they face barriers to integration, including a costly and time-consuming medical registration process [9,10,11,12,13].

Several promising local, regional and national initiatives aim to support the integration of Dutch status holders into healthcare professions [14,15]. However, many of these initiatives are not embedded within the health professionals training infrastructure of Dutch University Medical Centres, which are responsible for the formative training of doctors both pre-qualification and post-qualification in residency programs. As a result, existing programs fail to meet the demand among refugee health professionals, underscoring the need for sustainable, integrated curricula that can serve a broader range of newly arrived professionals.

## Goal of the Innovation

The goal of this innovation was to develop a bridging program that connects the potential of newcoming healthcare professionals with employment opportunities in the Netherlands. For this design, the term *‘status holders’* has been replaced with the more inclusive and welcoming label *‘newcomers’* (Table 1). The Newcomer program was developed within a tertiary university medical center in partnership with affiliated regional hospitals, using a design-based research (DBR) methodology [16,17]. DBR is a stepwise approach to create educational interventions for existing needs. This innovation involved multiple stakeholders, further described under step 2, 6 and 7 below, in close interaction and transdisciplinary collaboration. The mixed methods approach used allows evaluation and refinement of the innovation in continuous conversation with all partners. Running the program several times within the process provided better understanding of the most important learning goals and allowed room to analyze theories used while adding other models, in working towards the final version.

## Steps for Innovation Development and Implementation

**Figure 1:**
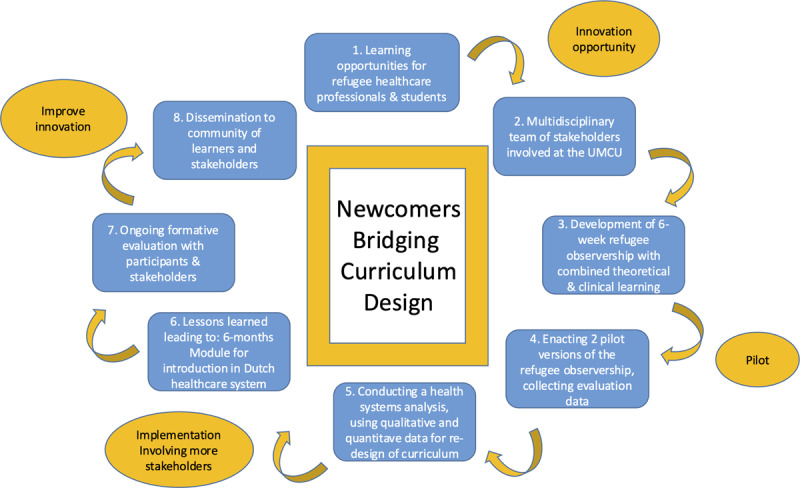
The eight steps of Design-Based Research in Medical Education, applied to Newcomers Bridging Curriculum Design.

The eight steps of DBR [16] were applied to guide the development, implementation and reporting of the Newcomer program between 2022 and 2024 and further improvement of the innovation in 2025 (Figure 1).

### 1. Define Opportunities

Following the escalation of the Ukrainian-Russian war in spring 2022, the University Medical Centre Utrecht (UMCU) received numerous requests for education and training opportunities from refugee healthcare graduates and students [18]. In response, an educational summer program was developed for refugees with (para)medical backgrounds to create an opportunity to continue their medical learning and development of knowledge, skills and attitude. Eligible participants for this initiative were diverse: refugees and status holders from all nationalities who were seeking to continue their education, complete their home-country medical degrees through supplementary ECTS credits, or enter the Dutch healthcare workforce with support for integration.

### 2. Involve Stakeholders

A dedicated multidisciplinary team of healthcare, education and policy staff at the UMCU collaborated to engage relevant internal stakeholders. The final team consisted of directors of the Graduate School of Life Sciences, the Faculty of Medicine, the vice dean of education, a policy advisor internationalization, international officers, (clinical) teachers, researchers, medical students, a healthcare system professional, and attending physicians from various disciplines.

### 3. Design & Develop

Between April 2022 and July 2022, the first version of the program was developed, informed by instrumental theories of cognitive and experiential learning [19], in the medical context of the UMCU: it was the refugee observership course. This six-week English language program combined theory based education and medical practice experience, focusing on nearly or recently graduated refugee medical students from diverse backgrounds. It consisted of two components: enrolment in one of UMCU’s existing global health summer schools, followed by short clinical rotations in different hospital departments combined with teaching sessions. This observership course served as a rapid response to urgent educational demands from medical refugees in the Netherlands. It aimed to familiarize participants with the Dutch healthcare system, continue their medical education and provide professional networks and a sense of belonging to their new country of residence. Where applicable, it also enabled participants to obtain outstanding European Credit Transfer and Accumulation System (ECTS) credits to finalize their degree-qualifying program in their country of origin. The program was accredited for 20 ECTS, enabling formal recognition by participants’ home universities and contributing towards their degree requirements.

### 4. Pilot

The first refugee medical observership took place in summer 2022. Following the course announcement, the program received an overwhelming number of applications for the limited eight places. To extend learning opportunities, eight additional spots were created for the classroom-based component of the course, as the main constraint was the limited availability of clinical placements. The working language was English. Participants were third-country nationals from Nigeria, Zambia and Syria who had been studying medicine in Ukraine and participants who had received medical training in Ethiopia, Malaysia, Afghanistan and Libya. Key lessons from the pilot included, beyond a confirmation of the high demand and strong internal motivation of candidates, a clearer understanding of the group’s needs in the Dutch language, and competency and skills training from an educator’s perspective. In the evaluation of this program (elaborated elsewhere (18)), the importance of practical support measures during the observership, including housing during the course, modest stipends, mentorship and peer reflection on cultural differences were also identified as critical for future programs.

### 5. Analyze

The encouraging evaluations from both participants, organizers and educators, combined with continued high demand, resulted in a second refugee observership in 2023. This second iteration confirmed that an academic healthcare setting serves as a suitable environment for experiential learning in a transcultural and international setting, as different medical departments showed a willingness to host these diverse students and switch language throughout clinical work from Dutch to English. However, it was evident that for sustainable integration and employment of settled refugees into the Dutch healthcare system, the language barrier would need to be further addressed. This second observership also confirmed that the academic surrounding was a critical steppingstone for refugee healthcare professionals’ careers in two ways: where applicable, it allowed them to obtain their diplomas, and by familiarizing them with the Dutch healthcare system, to prepare them for their next career step [18].

Thus, the two pilot editions of the refugee observerships underscored the urgent need for a more sustainable, comprehensive and scale-up follow-up program to improve foreign healthcare graduates’ integration into the Dutch healthcare system.

To design this bridging curriculum, a health systems analysis was conducted, comprising a desk review of relevant reports and articles combined with more than 20 formal conversations with key informants within the region. The review intended to identify stakeholders for potential collaboration. Key informants were purposely selected based on their experience with (refugee) healthcare training or the relevance of the organization they represented. This analysis aimed to identify barriers, success factors and best practices.

As this project fell under routine quality improvement and educational care innovation, no ethical research committee application was initiated.

**Figure 2:**
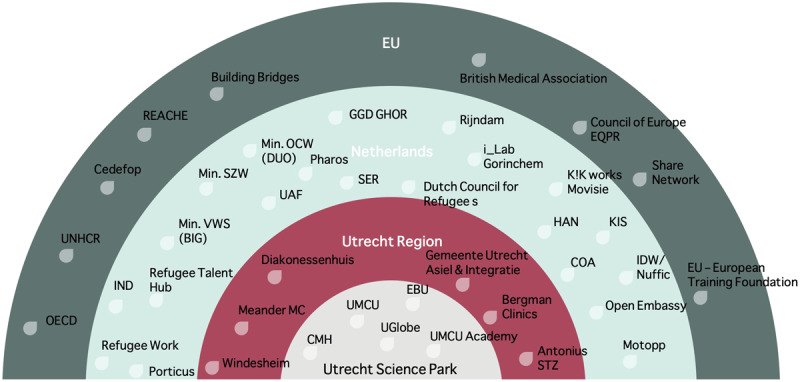
Overview of stakeholder organizations for newcomers’ support in the city of Utrecht, Utrecht region, the Netherlands and the EU. Legend of used abbreviations (for more detailed glossary, see appendix A): EU: European Union; OECD: Organization for Economic Cooperation and Development; UNHCR: United Nations High Commissioner for Refugees; REACHE: Refugee and Asylum Seekers Centre for Healthcare Professionals Education; IND: *Immigratie en Naturalisatie Dienst* Immigration and Naturalization Service; Min VWS (BIG): Ministry of Health, *Beroepen Individuele Gezondheidszorg* Professions in individual healthcare registration; Min SZW: Ministry of Social Affairs and Employment; Min OCW (DUO): Ministry of Education, Culture and Scientific Research*, Dienst Uitvoering Onderwijs* Executive organization for educational regulations; UAF: University Asylum Fund; SER: *Sociaal Economische Raad*, Social and Economic Council; HAN: *Hogeschool Arnhem en Nijmegen* Higher School of Arnhem and Nijmegen; KIS: *Kennisplatform Inclusief Samenleven* Platform Inclusion and Community; COA: *Centraal Orgaan Opvang Asielzoekers* Central Agency for the Reception of Asylum Seekers; IDW: *Internationale Diploma Waardering* International Qualification Acknowledgement; CMH: Central Military Hospital; UMCU: University Medical Center Utrecht; UGlobe: Utrecht University Center for Global Challenges; EBU: Economic Board Utrecht.

The quantitative and qualitative health systems analysis resulted in:

1. A broad stakeholder mapping (Figure 2; Appendix A, Figure 1) and -selection (Appendix A, Table 1), to identify institutional stakeholders at the local, regional, national and European level who could serve as partners, enablers or funders. The specific regional stakeholders in the Dutch healthcare system were selected based on two criteria: [1] whether the organization would benefit from improved integration of healthcare employees/students with a migration background, and [2] whether it could offer supportive funding.
2. Identification of barriers and good practices in the Netherlands and Europe based on formal conversations with status holders who started with a job or a study at the UMCU, and employers and resettled professionals with medical registration experience within the region (Appendix B). These conversations revealed success-enabling factors, barriers to integration, and high-potential quick wins.

Key success factors for healthcare integration included: language requirements (for enrolment into training at least level B1, for work readiness B2 or C1, plus at least one course in medical Dutch), housing within 45 minutes from work/studies, training in Dutch healthcare culture and practices, stability in the home situation (e.g. childcare and finances), a sense of belonging and access to reflection, peer reflection (intervision) and mentorship. Furthermore, hosting departments reported increased job satisfaction and appreciation for the new insights and cross-cultural understanding fostered through newcomer integration.

### 6. Implement

**Figure 3:**
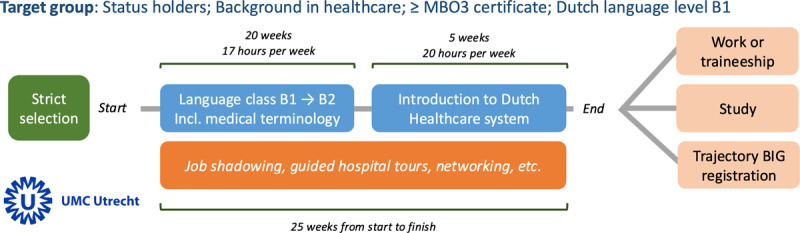
Overview of UMCU Newcomers Module for Introduction in Dutch Healthcare. **Legend:** MBO3: Tertiary vocational education level 3; Language level B1: Intermediate language user according to Common European Framework of Reference for Languages, ranging from A1 to C2, B2 being upper intermediate level; BIG: ‘*Beroepen Individuele Gezondheidszorg*’, Professions in Individual Healthcare qualification recognition. **Content of the Newcomers Module for Introduction in Dutch Healthcare:** -Intercultural communication and feedback -Dutch medical language up to B2 level -Dutch habits and culture in healthcare -Introduction in Dutch healthcare practice -Introduction in healthcare service provision, reimbursement and insurance -Personal guidance, reflection and group intervision -Practical support (resume, network, employment).

Insights from the two refugee medical observerships and the health systems analysis led to a re-design of the Newcomers bridging curriculum. This resulted in a 6-month program for newcoming healthcare professionals within the UMCU, titled ‘*Newcomers Module for Introduction in Dutch Healthcare’* (Figure 3). This module accommodated 12 participants per cycle. Participants were selected based on strict criteria: Dutch language skills (at least level B1), vocational training level (at least ‘MBO 3’, being 3 years of completed vocational training) and motivation.

New stakeholders in the program’s delivery included a regional Dutch language school and the municipality of Utrecht. In addition, clinical teams hosting newcomers received training aimed to create openness and foster cultural diversity.

Dutch medical students were involved in the module to facilitate newcomers’ integration, acting as ‘buddies’ familiarizing newcomers with the academic environment and creating opportunities for collaboration in joint projects, mutual learning, ‘internationalization at home’ and the development of intercultural competencies.

### 7. Evaluate

The first module supporting newcomer integration into the Dutch healthcare system was conducted between January 2024 and July 2024. Of the 83 applicants, 11 participants were selected. Most were based in the Utrecht region and had diverse healthcare backgrounds.

All participants completed the course reaching Dutch language level B2 or C1. Following the program, six participants secured employment in healthcare: two in the UMCU, two in participating regional hospitals, one in a healthcare clinic, and one at a physical therapy practice. One participant switched careers to daycare. The others continued studies and the qualification recognition process for their medical diplomas. Further research exploring participants’ perspectives on the module is ongoing.

In January 2025, the second edition of the module was announced and received 100 applications, primarily from the Utrecht region. The module began in April 2025. This edition involved regional stakeholders including two regional hospitals as partners for training and employment (Appendix A, Table 1). These hospitals collaborate in delivering lectures, mentoring participants, and preparing clinical teams for newcomer integration and intercultural engagement.

### 8. Disseminate

Dissemination of program outcomes and participant experiences to the community of (potential) learners and stakeholders occurred both internally and externally. Internal communication took place via the UMCU and partner institutions’ channels. External outreach included the UMCU’s website, social media, the annual report, regional newspaper articles and a national medical magazine [20].

## Outcomes of Innovation

This design-based research process revealed crucial components for a bridging curriculum aimed at newcomers’ integration in healthcare both within an academic hospital and at a regional level. The instrumental learning theories chosen initially have been extended with reflective models, consisting of personal guidance, group reflection and intervision, for the final curriculum design [19]. Both dialogue-based evaluation with pilot candidates and key informants’ conversations showed strong internal motivation of participants, but also their basic needs in terms of practicalities, belonging and mentoring (Appendix B). The resulting module combined a safe and effective learning environment with intensive medical language instruction, structured exposure to the Dutch healthcare system and culture and personalized guidance towards entry-level employment, internships or further study. It enabled participants to become familiar with healthcare culture, explore job and education opportunities and understand qualification requirements.

## Critical reflection on the process

Our tertiary academic hospital and regional stakeholders enabled us to combine organizational strengths in patient care, education and research to design a sustainable bridging pathway that helps newcomers overcome barriers and access healthcare employment and further education. The program responded to a growing demand, evident in the number of applicants exceeding available places, and the rapid interest from two regional hospitals to join the program.

The DBR process for this curriculum development, implementation and evaluation provided actionable insights in learning (pre)conditions for medical education for newcomers. We found that participants mainly expressed ‘physiological, safety and belonging needs’, being the basis of the hierarchy of needs according to Maslow [21]. Ensuring these preconditions are met was key in this innovation, through nearby and stable living situations, small stipends and support to find funding, a sense of belonging and access to reflection, intervision and mentorship. Language and cultural barriers also needed to be sufficiently addressed, following the hierarchy of needs ‘to know and understand’, to ensure actual learning can start [21]. Furthermore, this innovation showed the effectiveness of reflective models adding to cognitive and experiential learning.

Equally important to designing a bridge program is ensuring a clear pathway to the next stage of the newcomers’ integration into healthcare. Building onto the bridge metaphor, a safe landing ‘on the other side of the bridge’ is essential, by structured guidance for newly arrived professionals over one to two years. Accomplishing this goal may include ongoing group coaching, individual mentoring, peer reflection and intervision for program graduates. Further development of such a support strategy is recommended for future programs.

Successful integration of newcomers also requires flexibility and adaptability from clinical departments, (clinical) educators and students. To foster inclusive learning environments, the need for additional guidance and patience when coaching non-native Dutch-speaking colleagues needs to be anticipated and accommodated. This coaching requires an investment in time, education and training of (clinical) departments, for example, in intercultural competency development [22]. Importantly, the benefits of these efforts can be mutual, as involving newcomers has a positive effect on clinical teams and increases diversity, which is often still limited in Dutch healthcare [23]. As we observed during Step 5 of the design process, teams reported increased job satisfaction, appreciation for new insights and greater mutual cultural understanding. These benefits, gained by embracing new perspectives that enrich standard practice and broaden team strengths, can be incorporated into wider equity, diversity, and inclusion (EDI) initiatives or competency-based training in medical (specialist) education.

The current demand for these programs exceeds the capacity of a single institution, and interest is likely similarly high in other regions. Therefore, there is a clear need to scale up the program nationally. This need motivated the integration of educational research into program design, implementation and evaluation, as this approach shares learning across hospitals and healthcare institutions in the Netherlands and the rest of Europe.

Given the persistent, substantial and growing healthcare workforce shortages in the Netherlands [24], the onboarding of status holders within healthcare is an additional effective and ethical way to reduce these shortages. The potential of which has been demonstrated by the successful and longstanding Refugee and Asylum Seekers Centre for Healthcare Professionals Education program in the UK [25].

In conclusion, with the joint effort of hospitals, medical faculties and their regional stakeholders, the potential for integrating motivated and skilled newly arrived healthcare professionals can be realized. A bridging curriculum that embeds training, practice and an enabling supportive environment, can contribute meaningfully to building more inclusive and equitable learning and working opportunities for foreign healthcare graduates.

## Additional Files

The additional files for this article can be found as follows:

10.5334/pme.1994.s1 Appendix A. Stakeholder mapping and -selection.

10.5334/pme.1994.s2 Appendix B. Qualitative data for Newcomers Bridging Curriculum Design.

## References

[1] UNHCR. Refugee Data Finder – Key Indicators [Internet]. 2025 [cited 2025 Jul 10]. Available from: https://www.unhcr.org/refugee-statistics

[2] OECD. International migration trends [Internet]. 2024 [cited 2025 Jul 10]. Available from: https://www.oecd.org/en/topics/international-migration-trends.html

[3] Socha-Dietrich K, Dumont JC. International migration and movement of doctors to and within OECD countries – 2000 to 2018 [Internet]. 2021 [cited 2025 Jul 10]. DOI: 10.1787/7ca8643e-en

[4] EUAA, OECD. Voices in Europe: Experiences, hopes, and aspirations of forcibly displaced persons from Ukraine [Internet]. 2024 Mar [cited 2025 Jul]. Available from: https://www.oecd.org/content/dam/oecd/en/publications/reports/2024/03/voices-in-europe_980eb33d/ae33637c-en.pdf

[5] van Riemsdijk M. Career rewind: professional trajectories of pharmacists with a refugee background. Globalisation Soc Educ. 2023 Jul 26;23(3):1–12. DOI: 10.1080/14767724.2023.2236581

[6] Centraal Bureau voor de Statistiek (CBS). Statushouders huisvesting en integratie [Internet]. Cohortonderzoek asielzoekers en statushouders – Asiel en integratie 2024. 2024 [cited 2025 Jul]. Available from: https://longreads.cbs.nl/asielenintegratie-2024/statushouders-huisvesting-en-integratie/

[7] Centraal Bureau voor de Statistiek (CBS). Arbeidsmarkt zorg en welzijn [Internet]. 2025. Available from: https://www.cbs.nl/nl-nl/dossier/arbeidsmarkt-zorg-en-welzijn

[8] Centraal Bureau voor de Statistiek (CBS). CBS Statline AZW [Internet]. 2025 [cited 2025 Jul]. Available from: https://azwstatline.cbs.nl/#/AZW/nl/

[9] Stichting voor Vluchteling-Studenten UAF. Van A naar BIG tot Zorgprofessional: Een onderzoek naar de BIGregistratie en loopbaan van gevluchte artsen, tandartsen en apothekers in Nederland 2005–2020 [Internet]. 2021 Oct [cited 2025 Jul 10]. Available from: https://www.uaf.nl/wp-content/uploads/2021/11/UAF_Onderzoeksrapport-zorgprofessionals_2021.pdf

[10] Stichting voor Vluchteling-Studenten UAF. Haal gevluchte zorgprofessionals uit de wachtkamer! Praktijkervaring opdoen met een vakgenoot als mentor [Internet]. 2024 May [cited 2025 Jul]. Available from: https://www.uaf.nl/wp-content/uploads/2024/06/24_008_UAF.011-Infosheet-werkgevers-in-de-zorg-WEB.pdf

[11] van Twillert M. Een studie in Oekraïne, gevlucht en nu bij ons op dood spoor [Internet]. Medisch Contact. 2023 [cited 2025 Jul]. Available from: https://www.medischcontact.nl/actueel/laatste-nieuws/artikel/een-studie-in-oekraine-gevlucht-en-nu-bij-ons-op-dood-spoor

[12] Adviescommissie voor Vreemdelingenzaken (ACVZ). Migratie en de Zorgsector – Cijfers over de arbeidsmarkt in de zorgsector en de arbeidsdeelname van migranten [Internet]. 2021 May [cited 2025 Jul]. Available from: ACVZ+cijferoverzicht+migratie+en+de+zorgsector_toegankelijk.pdf.

[13] Adviescommissie voor Vreemdelingenzaken (ACVZ). Van asielzoeker naar zorgverlener – Arbeidsdeelname van asielmigranten in de zorgsector [Internet]. 2021 May [cited 2025 Jul]. Available from: 20210519+Summary++(van+asielzoeker+naar+zorgverlener).pdf

[14] Zorg voor het Noorden. Nieuwkomers in hun kracht – Leer-werktraject voor nieuwkomers in Nederland [Internet]. [cited 2025 Jul]. Available from: https://zorgvoorhetnoorden.nl/traject-nieuwkomers/

[15] Somsen A, Bercula N, Toor M van, Neijenhuis M. Win-win: buitenlands gediplomeerden opleiden tot echocardiografist. De impact van het opleiden van nieuwkomers in de zorg. Med Contact. 2024 Oct 10. DOI: 10.1097/ACM.0000000000004601

[16] Novak DA, Hallowell R. Design-Based Research: A methodology for studying innovation in teaching and learning in medical education. Acad Med. 2022 Jul 1;97(7). 10.1097/ACM.0000000000004601 35044977

[17] Diana HJ, Dolmans M, Tigelaar D. Building bridges between theory and practice in medical education using a designbased research approach: AMEE Guide No. 60. Med Teach. 2012 Jan 17;34(1):1–10. DOI: 10.3109/0142159X.2011.595437 22250671

[18] Horenberg R, Hoekstra J, van Wijnbergen JW, Homan I, Huizenga F, Stortelder E, et al. Guidelines and tips for establishing dedicated co-created medical educational programs for refugee-students in a high-income country healthcare institution. MedEdPublish [Internet]. 2025 Apr 14 [cited 2025 Jul];15:17. Available from: https://mededpublish.org/articles/15-17/pdf

[19] David C, Taylor M, Hamdy H. Adult learning theories: Implications for learning and teaching in medical education: AMEE Guide No. 83. Med Teach. 2013 Sep 4;35(11):e1561–e1572. DOI: 10.3109/0142159X.2013.828153 24004029

[20] van Twillert M. Uitbreiding voor Utrechts onderwijsprogramma voor statushouders in de zorg. Med Contact. 2024 Oct 10.

[21] Maslow AH. A theory of human motivation. Psychol Rev. 1943 Jul;50(4). DOI: 10.1037/h0054346

[22] Stahl GK, Maznevski ML. Unravelling the effects of cultural diversity in teams: A retrospective of research on multicultural work groups and an agenda for future research. J Int Bus Stud [Internet]. 2021 Jan 18 [cited 2025 Jul];52(1):4–22. Available from: https://link.springer.com/article/10.1057/s41267-020-00389-9 33487775 10.1057/s41267-020-00389-9 PMC7812115

[23] Mulder L, Wouters A, Akwiwu EU, Koster AS, Ravesloot JH, Peerdeman SM, et al. Diversity in the pathway from medical student to specialist in the Netherlands: a retrospective cohort study. Lancet Reg Health Eur. 2023 Dec 1;35:100749–9. DOI: 10.1016/j.lanepe.2023.100749 37860636 PMC10583163

[24] Waasdorp GJ. Arbeidsmarkt Zorg in Cijfers 2023 [Internet]. Intelligence Group. 2023 Sep [cited 2025 Jul]. Available from: https://intelligence-group.nl/nl/resources/arbeidsmarkt-zorg-in-cijfers-2023/

[25] Awan A, Browne JL, Ibrahim A, Butler C, Drovandi A. Training refugee and asylum seeking healthcare professionals: an ethical approach to UK workforce challenges. BMJ. 2025 Feb 18;388:e080992. DOI: 10.1136/bmj-2024-080992 39965798

