# Supplementary material for: Bridging the Gap: Designing Medical Integration Curricula for Foreign Healthcare Graduates in the Netherlands
Source: Perspect Med Educ. 2026 Mar 18;15(1):270–8. doi: 10.5334/pme.1994 (PMC13004058; doi:10.5334/pme.1994)
Supplement: Appendix A. — Stakeholder mapping and -selection. [file pme-15-1-1994-s1.pdf]

APPENDIX A: Stakeholder mapping and -selection

A wide range of societal stakeholders exists for engagement to develop and implement a bridging program for status holders. More specifically, 50+ organizations have been identified in the Netherlands (and other EU countries) that focus on the integration of refugees in the (Dutch) labor market or in healthcare in specific.

- **Appendix Figure 1: Stakeholder mapping**
  Mapping of organizations in Utrecht, Utrecht region, the Netherlands and the EU

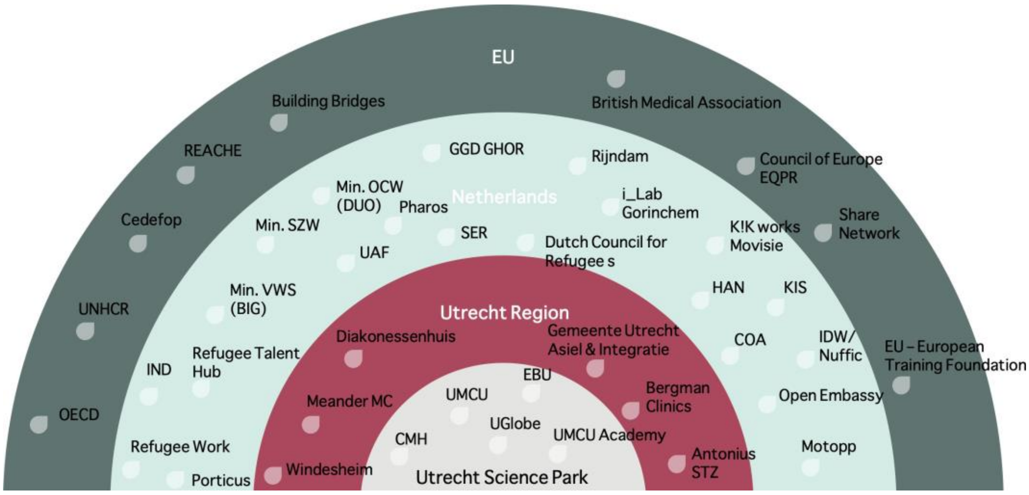

Appendix Figure 1: Stakeholder mapping

Glossary:

-<u>European Union (EU)</u>: OECD: Organization for Economic Cooperation and Development; UNHCR: United Nations High Commissioner for Refugees; Cedefop: European Centre for the Development of Vocational Training; REACHE: Refugee and Asylum Seekers Centre for Healthcare Professionals Education; Building Bridges: Redefining the EU's External Migration Agenda, European Policy Centre, co-funded by the EU; British Medical Association: Professional body for doctors in the UK; Council of Europe EQPR: European Qualifications Passport for Refugees; Share Network: Fostering inclusive communities for refugees and migrants in Europe; European Training Foundation: Dedicated to improving skills and employability of people in partner countries outside the EU

-<u>Netherlands:</u> Porticus: Philanthropic organization striving for a just and sustainable future by addressing social and environmental challenges; Refugee Work: Network of talents for job seekers and employers; IND: *Immigratie en Naturalisatie Dienst* Immigration and Naturalization Service; Refugee Talent Hub: Aims to connect refugee talent with work opportunities; Min VWS (BIG): Ministry of Health, *Beroepen Individuele Gezondheidszorg* Professions in individual healthcare registration; Min SZW: Ministry of Social Affairs and Employment; Min OCW (DUO): Ministry of Education, Culture and Scientific Research*, Dienst Uitvoering Onderwijs* Executive organization for educational regulations; UAF: University Asylum Fund; Pharos: National health center focusing on reducing health disparities; GGD GHOR: *Gemeentelijke Gezondheids Dienst* Municipal Health Service, *Geneeskundige Hulpverlenings Organisatie in de Regio* Medical Assistance in the Region during crises and emergencies; SER: *Sociaal Economische Raad*, Social and Economic Council; Rijndam: Focusing on rehabilitation care; Dutch council for refugees: Independent organization dedicated to supporting refugees and asylum seekers in the Netherlands; i_lab Gorinchem: Cooperative platform that brings together education, business and government to foster innovation and collaboration; K!X Works Movisie: Aims at assisting status holders in integrating into the Dutch labor market; HAN: *Hogeschool Arnhem en Nijmegen* Higher School of Arnhem and Nijmegen; KIS: *Kennisplatform Inclusief Samenleven* Platform Inclusion and Community; COA: *Centraal Orgaan Opvang Asielzoekers* Central Agency for the Reception of Asylum Seekers; IDW / Nuffic: *Internationale Diploma Waardering* International Qualification Acknowledgement, Dutch organization for internationalization in education, where one can apply for credential evaluation; Open Embassy: Focuses on supporting newcomers and facilitating their integration into the community; Motopp: Motivation Opportunity, Organization for helping newcomers gaining employment in the IT-sector

-<u>Utrecht Region</u> (being the province of Utrecht): Windesheim: Higher Education Institution; Meander MC: Meander Medical Center, Regional hospital; Diakonessenhuis: Regional hospital; Gemeente Utrecht, Asiel & Integratie: Utrecht Municipality, Refugees and Asylum Seekers integration; Bergman Clinics: network of specialized private medical clinics; Antonius STZ: *Antonius Samenwerkende Topklinsiche Ziekenhuizen*, Regional hospital, part of Collaborative Top Clinical Hospitals

-<u>Utrecht Science Park</u> (Innovative scientific community containing over 170 organizations and institutions within the city of Utrecht): CMH: Central Military Hospital; UMCU: University Medical Center Utrecht; UGlobe: Utrecht University Center for Global Challenges; EBU: Economic Board Utrecht; UMCU Academy: UMC Utrecht Young Academy, Group of researchers promoting an open and supportive research environment

- <u>Appendix Table 1:</u> **Stakeholder selection**
  Selection of possible relevant stakeholders in regional Dutch healthcare, for the development and implementation of a newcomer's integration curriculum design

Organization	Mission	Does organization benefit from good integration of employees/ students with a migration background in healthcare?	Potential funding for integration of employees/ students with a migration background in healthcare available?
**Antonius Hospital*** (Regional hospital)	Mission is to ensure quality of life, as a team	√	√
**Bergman Clinics** (Regional private clinic)	Mission is to deliver affordable, accessible and high-quality care, by attracting highly qualified personnel	√	√
**COA** (Central organ hosting asylum seekers)	Mission is to provide shelter or housing and guidance towards a future in the Netherlands	√	√
**Diakonessen Hospital*** (Regional hospital)	Mission is to deliver high quality, person-oriented care	√	√
**Dutch Council for Refugees**	Mission is to support and serve the interest of refugees and to support shelter, admission and participation in society	√	√
**Economic Board Utrecht**	Mission is to create economic progress in Utrecht region and to have a diverse group of individuals and businesses join Utrecht region, especially focusing on healthcare industries	√	√
**GGD GHOR** (Regional public health service)	Mission to strengthen public health in the Netherlands, focused on groups first before individuals	√	√
**Health Insurance Providers**	Mission is to create healthy circumstances for refugees as a main population that needs extra care resettling in a new country	√	√
**Meander Medical Centre** (Regional hospital)	Mission is to deliver people-oriented regional care	√	√

**Min. OCW** (Ministry of education, culture and science)	Mission is to work on a smart, skilled and creative Netherlands, where everyone can participate and where you have the freedom to be and express yourself	√	√
**Min. SZW** (Ministry of social affairs and employment)	Mission is to create equal job opportunities, healthy and safe working environments	√	√
**Min. VWS** (Ministry of health)	Mission is to support integration of status holders in healthcare jobs and if needed to deploy extra activities to enhance good integration	√	√
**Pharos**	Mission is to eliminate health discrepancies in certain groups in society; equal access to quality of care and access to a healthy lifestyle	√	√
**Porticus**	Mission to create a sustainable future where justice and human dignity flourish	√	√
**UAF** (University Asylum Fund)	Mission is to support refugees in making use of their knowledge; UAF is convinced that anyone who wants to make use of their skills is meaningful to the Netherlands	√	√
**Utrecht Municipality***	Mission is an inclusive, resilient and healthy city of Utrecht	√	√
*Stakeholder engaged in the final edition of the bridging program			

Appendix Table 1: Stakeholder selection
