# Supplementary material for: Bridging the Gap: Designing Medical Integration Curricula for Foreign Healthcare Graduates in the Netherlands
Source: Perspect Med Educ. 2026 Mar 18;15(1):270–8. doi: 10.5334/pme.1994 (PMC13004058; doi:10.5334/pme.1994)
Supplement: Appendix B. — Qualitative data for Newcomers Bridging Curriculum Design. [file pme-15-1-1994-s2.pdf]

APPENDIX B: Qualitative data for Newcomers Bridging Curriculum Design

Evaluation of best practices

Data have been obtained from 20 formal conversations with both status holders that started a job or education at the UMCU, as well as employers and resettled professionals. Among these purposively selected professionals were three with medical qualification recognition experience, being an emergency doctor from Latin America, a surgeon from the Middle East and a surgical assistant from Asia.

Summarized factors, out of best practices, either help or hinder good integration of newcomers in a job or study within the Dutch healthcare system, being:

(1) Success enabling
(2) Barriers to integration
(3) Potential quick wins

Best practices	How these factors help or hinder integration
**1. Success enabling**	
**1.1**<br>**Language level B2 or C1**	C1 language level* is preferred for good integration in medical departments and for small talk with patients.
**1.2**<br>**Dutch medical language (jargon)**	Being able to learn medical language (jargon) is highly recommended by resettling professionals.
**1.3**<br>**Housing in a 45 min. range**	A maximum 45 min. range from house to work or education is optimal.
**1.4**<br>**Sense of belonging**	By creating groups of max. 12 participants, offering an infrastructure for long-term group guidance (by forming protected groups on social media platforms like 'WhatsApp', or offering intervision), and by coordinating in-group help for questions or challenges – supported by accessible help from mentors and coaches from the organization.<br>By feeling welcome in departments for internships, feeling free to ask questions and to be given time to have a conversation in Dutch.<br>By receiving UMCU material, such as a note pad, a security card and hospital clothing.<br>Due to hardship and long stays in asylum seekers centers, participants might have a low sense of self-confidence. It is very helpful to cheer on, give trust and to emphasize they have every right to become a professional in the Netherlands.
**1.5**<br>**Expectation management**	By having a conversation *before* the participant starts language courses, medical schooling and registration for BIG** on what to expect.<br>This is especially important for highly trained medical professionals such as specialized surgeons. Chances are low that the same specialism can be obtained in the Netherlands, but they can be recognized as a doctor again.<br>It should also be emphasized that the qualification recognition process takes time and is costly (apart from enrollment, the internships are unpaid and leave many resettling healthcare professionals with taking a loan).<br>Training in the Netherlands/Europe for any medical profession is hard to compare to training for medical professions in other continents, so extra training is most likely applicable.
**1.6**<br>**Residence permit**	Registration, education and long-term jobs are only optional if a residence permit is assigned to the participant.

**1.7**<br>**Peer support**	See also 1.4.<br>In addition, a 'buddy' from a former program or a medical student acting as such, is highly valuable for resettling professionals and new students.<br>Some organizations offer a matching program with former healthcare professionals who now offer guidance to new or resettling healthcare professionals.
**1.8**<br>**Personal coaching**	When a participant starts integration in a Dutch healthcare organization, personal coaching should start and be continued on the long term. Coaching means to discuss intercultural experiences and experienced situations in their teams/departments or in their class that require extra perspective or nuance.<br>On a long-term basis, coaching aims to monitor mental health as the participant is resettling in a new country, job and environment.
**1.9**<br>**Mentorship and customized programs**	Each individual should be mentored intensively and customizable programs should be available, e.g. not having to do all internships, but just the ones you need.
**1.10**<br>**Strict selection and monitoring**	Programs and routing offered to participants should have strict selection (for example on language, travel distance etc.) in order to help the participant succeed. Other programs learned that if the commute is too long or there is e.g. no daycare available for children of the participant, the participant did not succeed.<br>In that case it is recommended to help someone with the next step, e.g. connect with municipality, and welcome them again for selection in a next round.<br>Monitoring: most successful integration programs have monitoring moments in which they review if the goals of the program and the presence are met, if not, strict regulations give the participant one warning, after that they have to leave the training.
**1.11**<br>**Having financial means**	Immigrating participants that have a good economic status, can invest in (language) courses, pay for the qualification recognition process and compensate for the loss of income during internships. This enhances getting a job in the Netherlands. It also brings a sense of inequality of which organizers should be aware.<br>In collaboration with Dutch municipalities and organizations like the UAF***, there should be options for newcomers to keep their financial support, while in training. As training requires intense time-investment and leaves little time to earn an income.
**1.12**<br>**Personality wise**	We asked professionals, colleagues, other programs and resettling professionals what personality traits help in successful integration in a healthcare job or education. Reported traits were having openness to new people and willingness to speak a new language even though it is at a basic or intermediate level.
**1.13**<br>**Strong support and expertise structure in organizations**	Organizations should be aware that welcoming colleagues with a culturally diverse background is a necessity to maintain our workforce and to increase quality of healthcare. Hosting departments reported increased job satisfaction and appreciation for the new insights and cross-cultural understanding fostered through newcomer integration.<br>This requires new organizational needs and expertise, reflected in an organizational structure. For example by a department that supports newcomers in their individual needs (extra language lessons, other training or career planning) and colleagues and managers that welcome newcomers in having an open view and maximizing the potential of diverse teams. This department could also focus on utilizing low-entry jobs as a career start. An in-house department that combines both educational and human resource expertise would be most suitable.

2. Barriers to integration	
**2.1<br>Intense language courses vs. theory combined with practical lessons**	Best result for learning the Dutch language is by enrolling in a low entry-level job that requires some speaking and listening in Dutch, supported by theoretic language lessons.<br>A crash course (for example a few weeks of fulltime language courses) has a good result in the short term, but the effects decreases in a few weeks after the course has ended.
**2.2<br>'Good spirit' is affected by long waiting time**	Some participants had to wait for years before they could (re)start their education or job. This could result in having difficulties adjust to a work or school system, requiring extra support.
**2.3<br>Unclear housing situation**	The Netherlands currently lacks housing for status holders, meaning many participants might still have to move around between asylum seekers centers or experience a move. This disturbs time and headspace to integrate in a job or education.
**2.4<br>Lack of intense monitoring**	If there is not enough time for a mentor to monitor the (mental) health of participants and to assess the home situation, the participant might discontinue education or working because there is no time left for dealing with the challenging situation. It is recommended to inform buddy's (from former programs or former healthcare professional-buddy's from a guidance program) that their help is desirable when it comes to monitoring if a participant is in need of extra (mental) support. It also needs regularly checks with all buddy's if there are signals that the team should keep an eye on.
**2.5<br>Commute**	Extra costs for public transport or other forms of transportation can cause a financial barrier.
**2.6<br>Personal circumstances**	Family and children that need care can consume a lot of time and energy.
**2.7<br>Personality traits**	Loneliness, no support for trauma or mental health and fear to speak a new language, cause personal barriers to integration.
**3. Potential quick wins**	
**3.1<br>Availability of psychosocial support**	Ensuring psychosocial support for example by the practice mental health worker of the general practitioner, to whom the mentor or coach can refer. Closely monitoring a healthy integration in the newcomer's new work- and living environment.
**3.2<br>Direct integration in a job**	Jobs that require no or limited Dutch language skills would be a great opportunity for status holders to feel a sense of belonging, learn the Dutch language and become familiar with the way of working in Dutch organizations, while being supported in creating next career steps.
**3.3<br>Long-term follow-up and guidance**	Create an infrastructure that offers two personal coaching sessions and two group intervisions on a yearly basis, to exchange experiences, discuss findings and tips and to create and maintain a sense of belonging. This could also serve as a space to discuss next steps if someone is ready for a new position.

*Language levels according to Common European Framework of Reference for Languages, ranging from A1 to C2. B2 being upper intermediate level, C1 being advanced level.

**BIG: '*Beroepen Individuele Gezondheidszorg*', Professions in Individual Healthcare qualification recognition

***UAF: University Asylum Fund. See also Appendix A; Stakeholder mapping and -selection
